# Gestational misalignment with fixed exposure windows: the potential dangers of zero-filling in distributed lag models

**DOI:** 10.1093/ije/dyag162

**Published:** 2026-09-08

**Authors:** Michael Leung, Andreas M Neophytou, Ander Wilson

**Affiliations:** Department of Environmental Health, Harvard T.H. Chan School of Public Health, Boston, MA, United States; Department of Environmental and Radiological Health Sciences, Colorado State University, Fort Collins, CO, United States; Department of Statistics, Colorado State University, Fort Collins, CO, United States

**Keywords:** air pollution, bias, distributed lag models, prenatal exposures, zero-filling

## Abstract

**Background:**

Distributed lag models (DLMs) are widely used in perinatal epidemiology to identify critical windows during pregnancy in which environmental exposures influence pregnancy/birth outcomes. A well-known complication of fitting DLMs in this context is that they require the same length of exposure history for every individual even though not all pregnancies are of the same duration. This misalignment often leads researchers to artificially extend exposure histories to a fixed length and fill post-birth weeks with zeroes (i.e. “zero-filling”). Despite its widespread use, the implications of zero-filling have not been formally evaluated.

**Methods:**

We demonstrate conceptually that zero-filling induces a spurious association between gestational age and late-pregnancy exposures, thus introducing confounding that was otherwise not present in the observed data. We then conducted a simulation study and a real data application using air pollution and birth weight data from a Colorado-based cohort to compare zero-filling with alternative approaches to handle the misalignment between exposure window and gestation.

**Results:**

In our simulations, we found that zero-filling produced the largest bias, poorest coverage, and highest root mean squared error. In the analysis of the Colorado birth data, zero-filling produced implausibly strong associations. Adjusting for gestational age attenuated this bias. Alternative approaches of carrying forward the last pre-birth exposure value, using observed post-birth exposures, and, in some situations, truncation at 37 weeks eliminate this bias.

**Conclusion:**

Zero-filling can cause bias in the estimated associations when using distributed lag models.

Key Messages

- In perinatal epidemiology, a common approach to handling the misalignment between the length of exposure histories and the duration of pregnancy when fitting distributed lag models (DLMs) is “zero-filling”, whereby post-birth exposures are filled with zeroes.
- Our simulations and real data application using Colorado birth records show that zero-filling is conceptually problematic, as although it implicitly assumes that newborns experience no pregnancy exposures after delivery, it changes the structure of the exposure time series in a way that can bias the DLM estimates.
- We provide several alternative approaches for dealing with misalignments between exposure windows and gestation that are not prone to the same type of bias inflicted by zero-filling.

## Introduction

Distributed lag models (DLMs) are widely used to investigate delayed health effects [1]. When investigating the environmental determinants of pregnancy/birth outcomes (e.g. preeclampsia, birth weight), DLMs are often employed to identify potentially relevant prenatal windows in which the exposure of interest (e.g. air pollution, temperature) may be associated with the outcome [2–4]. For example, if we were interested in examining which gestational weeks fine particulate matter (PM_2.5_) may be associated with birth weight, then we could fit the following DLM:


$$y_{i}=\sum_{t=1}^{T}a_{it}\theta_{t}+L_{i}^{\prime }\beta +\varepsilon_{i},$$

where $y_{i}$ is birth weight for newborn i, $a_{it}$is the PM_2.5_ exposure in gestational week t, $\theta_{t}$ is the linear association between PM_2.5_ in week t and the outcome, $L_{i}^{\prime }$ is a vector of baseline covariates with corresponding coefficients β, and $\varepsilon_{i}$ is the error term. To reduce the effects of multicollinearity among repeated measures of exposure, DLMs are constrained, most commonly with a natural spline, so that the lag–response function $\theta_{1},\ldots ,\theta_{T}\;$varies smoothly over time. Note that in most DLM applications, exposures are lagged backward in time relative to the outcome, but in perinatal epidemiology, the temporal structure is often reversed, whereby exposures are indexed forward from conception so that each lag represents a gestational week.

A well-known but often-overlooked complication of fitting DLMs in perinatal research is that they require the same length exposure history for every observation even though not all pregnancies are of the same length (i.e. weeks 1 to T in the above DLM). Thus, researchers are required to perform data engineering steps to standardize the lengths of exposure histories and, in doing so, often create a mismatch between the length of exposure histories used in the model and the duration of pregnancy. For time-to-event outcomes, there are ways to deal with this misalignment—e.g. by fitting separate DLMs for each risk set [5] or by fitting separate models for each exposure week [6, 7], and then combining the estimates afterwards—but they are computationally intensive and are not always feasible. For other outcomes with no time component (e.g. birth weight), there is no established method, and so if the misalignment is not handled correctly, then it could lead to biased results.

A common approach to handling this misalignment is “zero-filling,” whereby everyone’s exposure history is extended to a fixed number of weeks regardless of when they are born (e.g. 42 weeks), and then post-birth exposures are filled with zeroes [8–13]. This practice is typically justified as a pragmatic solution to mismatched exposure and gestational timelines, where the motivation is to introduce “null” effects for lag times that are not contributing to the overall risk. For many exposures like air pollution, the logical value is zero (i.e. no exposure if no longer prenatal), but for other exposures such as temperature which display nonlinear health associations [14], there are no natural null values, and so this practice is not always applicable.

The consequences of zero-filling have not been formally evaluated. In this paper, we discuss why filling post-birth exposures with zeroes is conceptually problematic—although it implicitly assumes that newborns experience no pregnancy exposures after delivery, it changes the structure of the exposure time series in a way that can bias the lag–response estimates. We conduct a simulation study and a real data application to empirically illustrate the impact of zero-filling on inference when using DLMs and compare zero-filling to alternative approaches for dealing with misalignments between exposure windows and gestation. We then offer practical recommendations for dealing with varying gestational ages in DLM analysis of birth outcomes.

## Development and application

### Bias due to zero-filling

**Figure 1:**
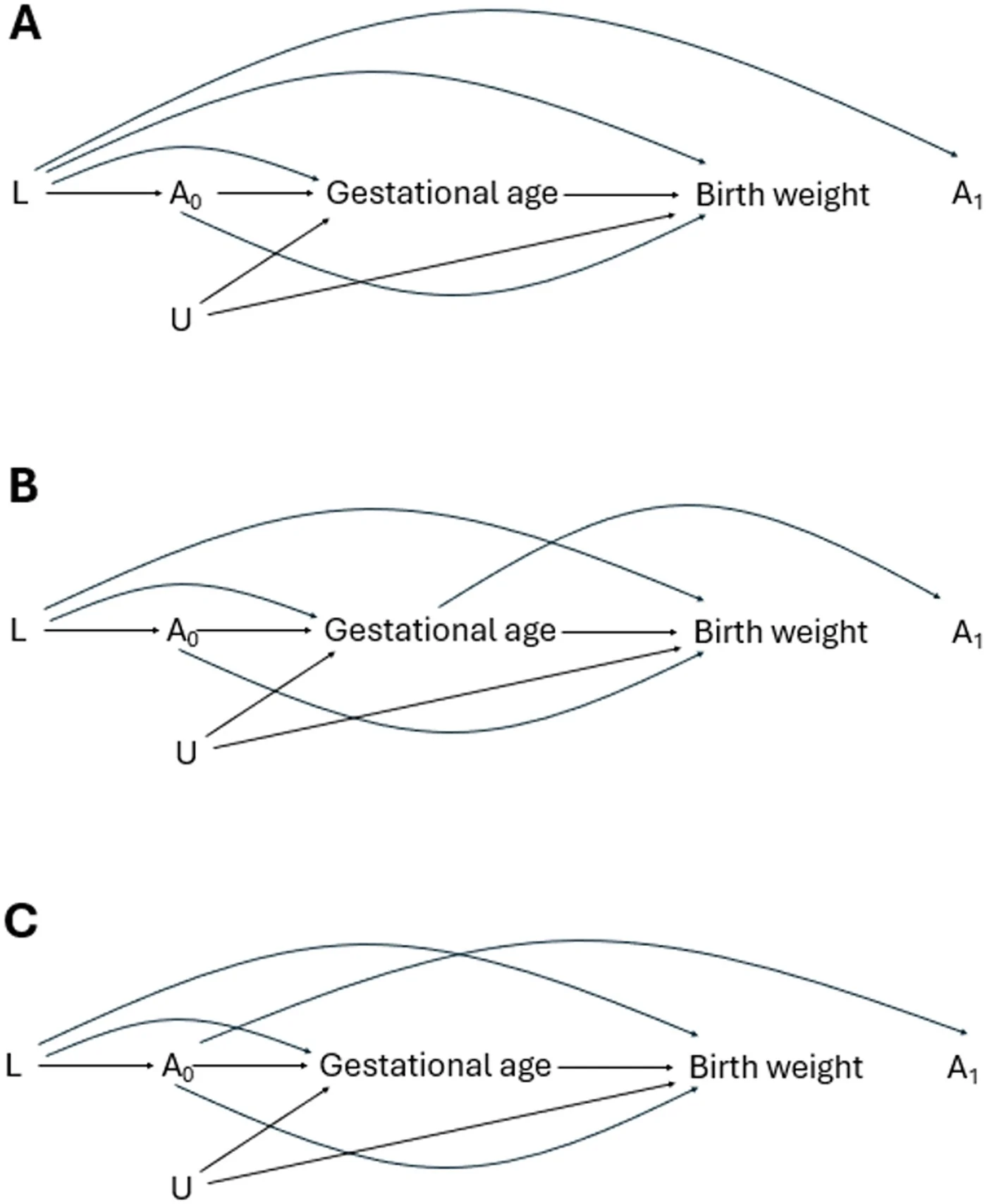
Directed acyclic graphs (DAG) of different approaches to handling exposure and gestational misalignments when examining the association between air pollution and birth weight, where $A_{0}$ is pregnancy exposure, $A_{1}$ is post-birth exposure, L is a common cause of air pollution and birth weight, and U is a common cause of gestational age and birth weight. DAG (A) represents the structure of using their natural exposure, (B) represents the structure of zero-filling, and (C) represents the structure of mean-filling or carrying forward.

We first outline the structural source of the bias from zero-filling using directed acyclic graphs (DAGs) as shown in Fig. 1. Consider the association between PM_2.5_ and birth weight. If we used residential addresses to assign pregnancy exposures $A_{0}$ and post-birth exposures $A_{1}$, which we will refer to as their “natural exposure” (Fig. 1a), then $A_{0}$ and $A_{1}$ are correlated based on the characteristics of where they live as well as when they lived there (i.e. space and time), which we denote by vector L. Thus, if we adjust for the appropriate set of area-level variables (e.g. area-level socioeconomic status, urbanicity) and time trends (e.g. seasonality, long-term trends) to block the backdoor path through L, then including post-birth exposures $A_{1}$ in the DLM should not bias the estimates for the effect of pregnancy exposures $A_{0}$ on birth weight, as they should be conditionally independent.

Now consider the practice of zero-filling as depicted in Fig. 1b. Because post-birth weeks are populated with zeroes and not the exposure they would have experienced at their residential address, $A_{1}$ is no longer determined by where they live (i.e. no $L\to A_{1}$ arrow). Instead, it is solely determined by the duration of pregnancy, whereby individuals with longer pregnancies have fewer zero-filled weeks and so will, on average, experience higher exposures. For example, if we consider a 42-week exposure window, then a child born in week 30 will have weeks 31–42 filled with zeroes, while the one born in week 40 will only have weeks 41–42 filled with zeroes. In this scenario, the 30-week birth will have a lower average exposure in the later stages of the exposure window (because of more zeroes) than the 40-week birth. In a DAG, this is akin to drawing a new arrow from gestational age to $A_{1}$, which creates a confounding path ($A_{1}\leftarrow \mathrm{gestational}\;\mathrm{age}\to \mathrm{birth}\;\mathrm{weight}$). We would expect the direction of this bias to be in the upward direction since gestational age is positively correlated with both birth weight and $A_{1}$. To remove the bias due to zero-filling, one may instinctively condition on gestational age in the analysis (e.g. stratification, z-scores), but this could block the indirect effect of $A_{0}$ (through gestational age) as well as induce collider-stratification bias by opening up a non-causal path through U, which is a common cause of gestational age and birth weight ($A_{0}\to \mathrm{gestational}\;\mathrm{age}\leftarrow U\to \mathrm{birth}\;\mathrm{weight}$) [15–17].

A widely used alternative to zero-filling is to restrict the exposure to a common exposure window (“truncating”). For example, if the shortest pregnancy in our cohort was 37 weeks, then we could fix the exposure window to 37 weeks and exclude exposures post-37 weeks. This would function to remove $A_{1}$ from the DAGs depicted in Fig. 1. In theory, this is a valid approach if the effect of the exposure is early in pregnancy, as the omission of exposures after 37 weeks should not cause any bias. For example, when investigating the effect of traffic pollution on pregnancy loss, the critical exposure window was found to be gestational weeks 8–20 [3]. If we truncated the exposure to as short as week 21, then we would still be able to identify this critical window without any bias. However, if there was an effect later in pregnancy (i.e. after week 21), then restricting the exposure to 21 weeks does not allow us to identify this effect (since the DLMs do not extend that long), and it could also distort the critical window as exposures before and after week 21 are correlated [2].

Other potential approaches include filling post-birth exposures with the mean air pollution level during pregnancy (“mean-filling”) or the exposure value from the last week of pregnancy (“carry forward”). These approaches are not expected to generate any bias, as post-birth exposures are no longer determined by gestational age but rather by some function of air pollution levels experienced during pregnancy. This can be represented as an arrow from $A_{0}$ to $A_{1}$ in the DAG depicted in Fig. 1c. It is important to note that because this is akin to setting $A_{1}$ to $A_{0}$, where gestational age itself has no role in setting $A_{1}$, there is no direct arrow from gestational age to $A_{1}$. Unlike zero-filling, as long as both $A_{0}$ and $A_{1}$ are included in the DLM, then they should be conditionally independent (i.e. there is no backdoor path from $A_{1}$ to birth weight conditional on $A_{0}$).

### Monte Carlo simulation

To numerically illustrate these results, we conducted a Monte Carlo simulation study. We simulated 1000 births with gestational ages from 37 to 42 weeks. For each birth, we randomly selected a 42-week exposure history from the Colorado birth registry linked to weekly average PM_2.5_ exposure data from the US Environmental Protection Agency fused air quality surface downscaler data [18] (see Supplementary Section S1 for more details on the Colorado data). We consider three data-generating scenarios: (A) gestational age is a predictor of birth weight with no mediation and no confounding; (B) gestational age is a mediator between exposure and birth weight; and (C) same as scenario A with the addition of known confounders (see Supplementary Section S2 for more details on these scenarios).

We compared five methods to align exposure histories: (i) post-birth exposures were filled with zeros (zero-filled); (ii) post-birth exposures were filled with the mean exposure from conception through birth for that dyad (mean-filled); (iii) post-birth exposures were filled with the exposure level for the birth week (carry-forward); (iv) using exposure in weeks 1–42 for all and including post-birth exposures (natural exposure through week 42); and (v) using exposure in weeks 1–37 following conception regardless of birth week (truncated at week 37). For each simulated dataset and data alignment method, we estimated a DLM with a natural spline basis. We considered degrees of freedom (df) ranging from 3 to 8 and selected the df for each dataset and alignment method using the Akaike information criterion. We used a quadratic term to adjust for gestational age at birth, and we also considered models with no gestational age adjustment. We simulated 2000 datasets and evaluated those using root mean squared error (RMSE) and coverage for each week and for the cumulative association defined as the sum of week-specific effects. We also evaluated bias for the cumulative effect.

In secondary simulations, we lowered the gestational age threshold from 37 to 30 weeks to examine the impact these methods have when births occur earlier. Our R syntax for the simulations can be found at https://github.com/AnderWilson/ZeroFillDLM.

**Figure 2:**
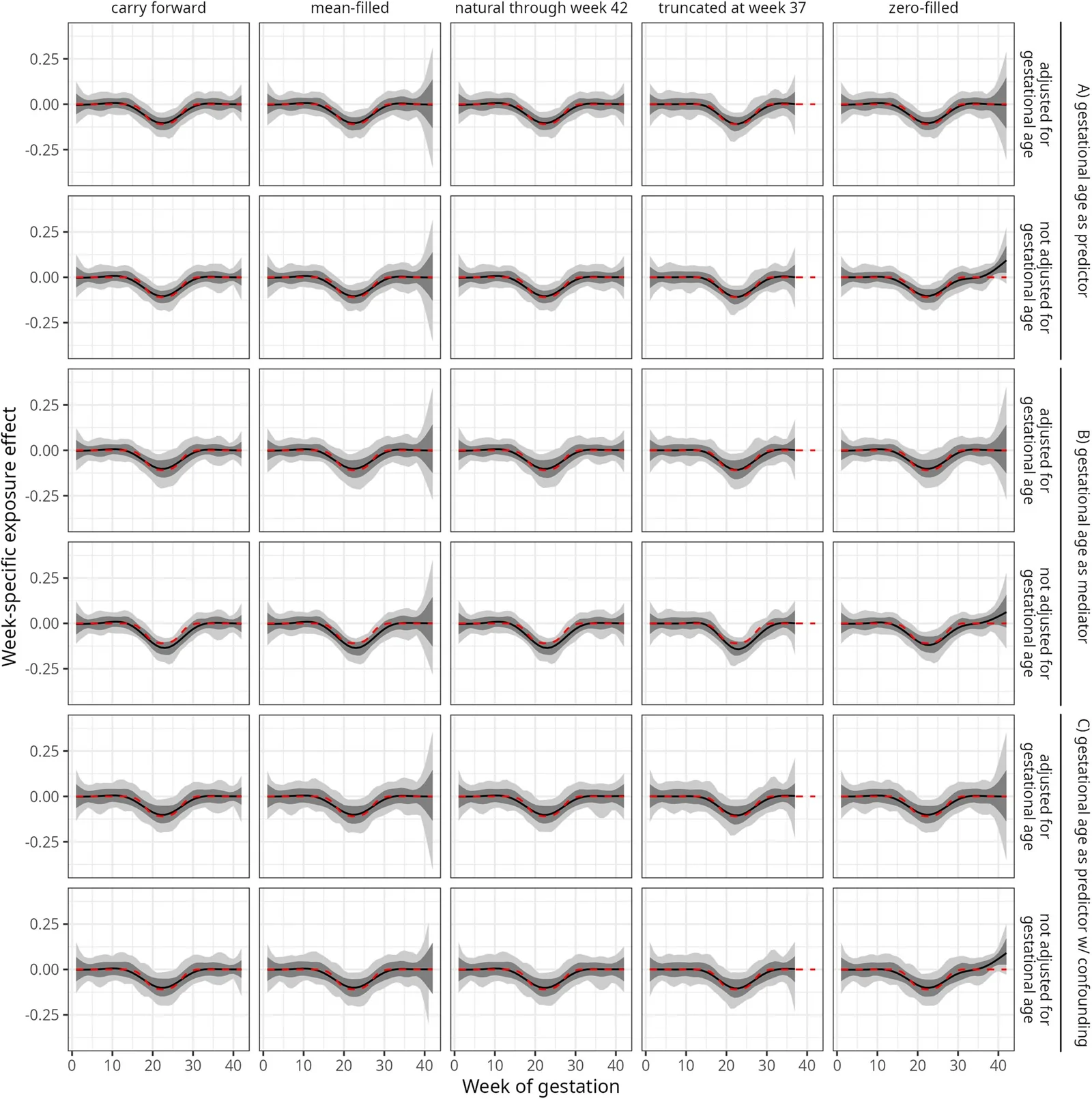
Simulation results showing the time-varying association between fine particulate matter (PM_2.5_) and birth weight for five approaches to handling exposure and gestational misalignment (carry forward, mean-filled, natural through week 42, truncated at week 37, and zero-filled). Each column of panels contains results using one data method. The rows of panels contain different scenarios (labeled A, B, and C) and models adjusted for or not adjusted for gestational age at birth. The dashed red line indicates the true simulated lag–response relationship. The solid black line shows the mean of 2000 simulated data sets. The dark gray ribbon shows the 0.05–0.95 quantiles of the week-specific estimates, and the light gray ribbon shows the minimum and maximum of the week-specific estimates.

**Figure 3:**
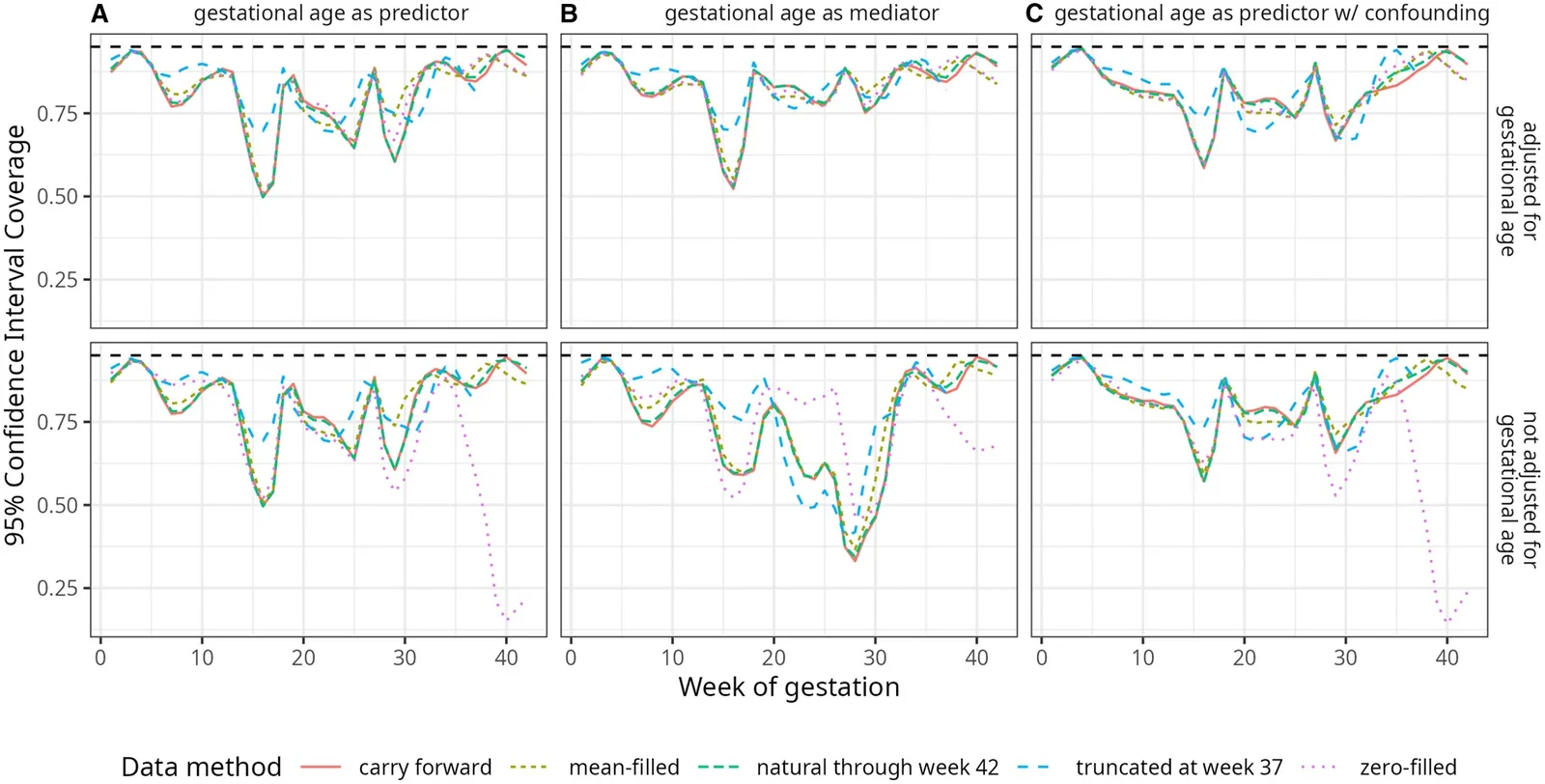
Simulation results showing the time-varying coverage between fine particulate matter (PM_2.5_) and birth weight for five approaches to handling exposure and gestational misalignment (carry forward, mean-filled, natural through week 42, truncated at week 37, and zero-filled). The six panels represent three difference simulation scenarios (denoted by columns A, B and C) and models adjusting for or not adjusting for gestational age at birth (rows). Each line indicates the proportion of simulations in which the lag-specific 95% confidence intervals contained the true simulated effect for one of the data methods, and the black dashed line indicates the 95% nominal coverage.

**Figure 4:**
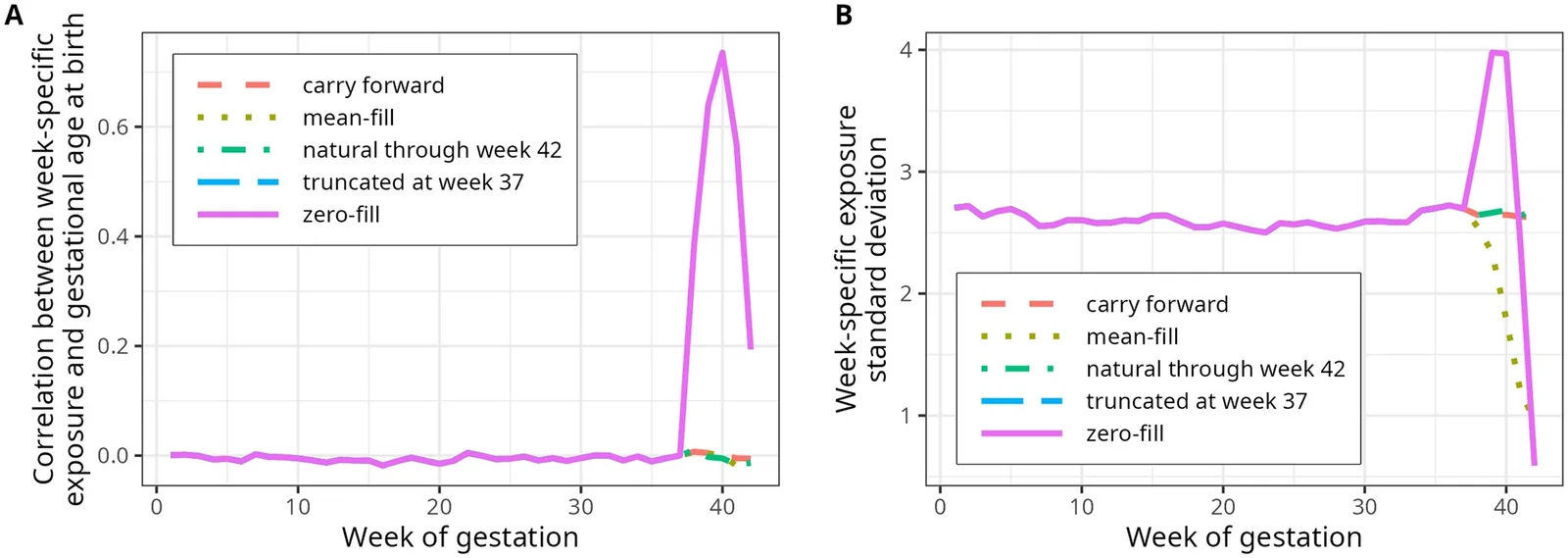
A comparison of five methods for handling exposure and gestational misalignment (zero-filled, carry forward, natural exposure through week 42, mean-filled and truncated at week 37) in terms of (A) the correlation between gestational age and weekly exposures and (B) the standard deviation of weekly exposures.

Bias, RMSE, and coverage for the cumulative association in each simulation are presented in Table 1. In scenario A, we found that zero-filling produced the largest bias (0.252) and RMSE (0.101), and the lowest coverage (70.4%) across all methods when not adjusting for gestational age. When looking at the lag–responses from the DLMs, the poorer performance of the zero-filled approach is mostly driven by the bias near the end of the exposure window (Figs 2 and 3). This is caused by the strong correlation between gestational age and exposure induced by zero-filling, which does not occur in other methods (Fig. 4). While unbiased, the mean-filling approach resulted in larger variance of the effect estimates in late weeks (Fig. 2). This is a result of lower variance in the exposure data for those weeks that are mean-filled (Fig. 4). In gestational age-adjusted models, the zero-filled method performed better than the unadjusted model (bias: −0.009, RMSE: 0.070, coverage: 92.4%), but still comparatively worse than the other methods.

**Table 1:** Simulation results showing bias, root mean square error (RMSE), and percent coverage of the cumulative association between air pollution and birth weight for the different approaches to handling exposure and gestational misalignment.^a^

	Not adjusted for gestational age			Adjusted for gestational age		
	Bias	RMSE^b^	Cover.^c^	Bias	RMSE^b^	Cover.^c^
(A) Gestational age as predictor						
Carry forward	−0.003	0.030	95.4%	−0.003	0.029	95.1%
Mean-filled	−0.003	0.030	95.0%	−0.003	0.029	95.2%
Natural through week 42	−0.001	0.031	94.8%	−0.001	0.031	94.8%
Truncated at week 37	−0.001	0.029	95.0%	−0.001	0.028	95.2%
Zero-filled	0.252	0.101	70.4%	−0.009	0.070	92.4%
(B) Gestational age as mediator						
Carry forward	−0.342	0.146	48.4%	0.016	0.068	93.4%
Mean-filled	−0.341	0.145	48.3%	0.019	0.068	92.8%
Natural through week 42	−0.340	0.146	50.3%	0.017	0.069	93.3%
Truncated at week 37	−0.340	0.144	47.6%	0.006	0.068	92.7%
Zero-filled	−0.015	0.089	92.4%	0.017	0.102	91.2%
(C) Gestational age as predictor w/confounding						
Carry forward	−0.006	0.037	95.5%	−0.006	0.037	95.1%
Mean-filled	−0.005	0.037	95.7%	−0.005	0.037	95.4%
Natural through week 42	−0.004	0.039	95.3%	−0.004	0.038	95.2%
Truncated at week 37	−0.006	0.037	95.3%	−0.005	0.036	95.3%
Zero-filled	0.235	0.099	76.6%	−0.006	0.076	92.8%

a The true cumulative effect in the simulated data is 1.

b Root mean squared error.

c 95% confidence interval coverage.

d Performance metrics computed for the direct effect (i.e. not through gestational age).

In scenario B, where we examined the performance metrics for the direct effect (i.e. not through gestational age), we found that for all methods except for zero-filling, there was a negative bias, as there is the additional indirect path mediated through gestational age which has not been accounted for (Table 1). However, we found that the positive bias from zero-filling counteracts this bias such that there is comparatively little bias and near-nominal coverage for the zero-filled method (bias: −0.015, RMSE: 0.089, coverage: 92.4%). In models adjusting for gestational age, we found that all methods performed similarly. In scenario C, we found that when we introduced covariate effects, the findings were similar to scenario A. Finally, when repeating the simulation settings, the minimum gestational age at 30 weeks, we found that the same pattern holds. However, the overall performance was unexpectedly better, but this may be because the splines are better at smoothing out the bias if it covered more weeks (Supplementary Figs S1, S2 and Table S1).

### Data application

**Figure 5:**
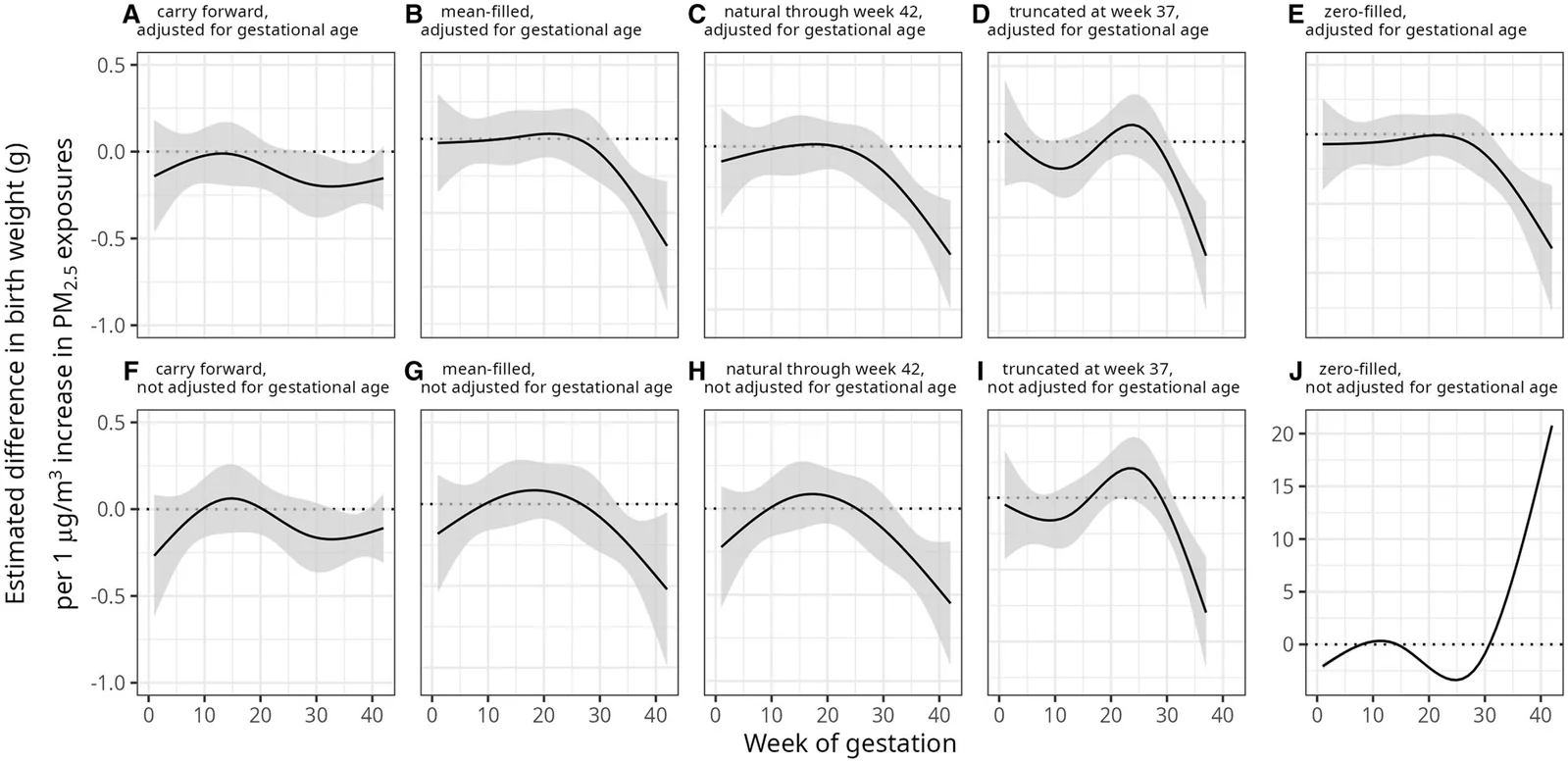
Results from the analysis of birth weight in the Colorado birth registry data (*n *= 393 204). The figure shows the week-specific association between PM_2.5_ exposure and birth weight using the five different data methods (columns) and adjusted for (panels A–E) and not adjusted for (panels F–J) gestational age (rows). The black solid line is the point estimate, and the gray ribbon is a pointwise 95% confidence interval. Panel J shows the estimates for the zero-filled data augmentation method without adjustment for gestational age and has a different *y*-axis scale than the other panels.

We fitted DLMs to the Colorado birth data to estimate the PM_2.5_-birth weight association under the five methods to align exposure histories with and without gestational age adjustment. All models were adjusted for known confounders described in Supplementary Section S1. In the primary analysis, we included only full-term births (born in weeks 37–42). In models that did not adjust for gestational age, we found an implausibly strong positive PM_2.5_-birth weight association in the zero-filled approach, where the change in birth weight was 76.01 g [95% confidence interval (CI): 73.03–78.98] per 1 µg/m^3^. This was driven by the strong positive week-specific estimates starting in week 30. In the other approaches, the cumulative association was negative (Table 2; Fig. 5). In gestational age-adjusted models, we found that all methods produced similar lag–responses where the cumulative association was more strongly negative. This is likely because the biasing forces have changed (e.g. there is now collider bias, which was not present before, and there is no longer confounding by gestational age if there are common causes of exposure and gestational age). Furthermore, we found that the CIs were tighter since gestational age is a strong predictor of birth weight, whereby it helps remove the extraneous variation in birth weight unrelated to PM_2.5_ (Table 2; Fig. 5). Results were similar in our secondary analysis, which included births starting in week 30 (Supplementary Fig. S3 and Table S2).

**Table 2:** Results from the analysis of birth weight in the Colorado birth registry data (*n *= 393 204).^a^

Approaches	Difference in birth weight (95% confidence intervals) (g)	
	Not adjusted for gestational age	Adjusted for gestational age
Carry forward	−3.55 (−6.70 to −0.41)	−4.73 (−7.64 to −1.82)
Mean-filled	−3.69 (−6.85 to −0.53)	−5.07 (−7.99 to −2.15)
Natural through week 42	−4.58 (−7.80 to −1.35)	−5.99 (−8.98 to −3.01)
Truncated at week 37	−3.44 (−6.56 to −0.32)	−4.50 (−7.39 to −1.61)
Zero-filled	76.01 (73.03 to 78.98)	−7.28 (−10.54 to −4.02)

a The table shows the estimated cumulative association (95% confidence intervals) for each data augmentation method and for adjusting for and not adjusting for gestational age at birth. The cumulative association is the expected difference in birth weight (g) per 1 μg/m^3^ increase in PM_2.5_ for all 42 weeks (or 37 weeks for the truncated model).

## Discussion

In this paper, we demonstrate conceptually and through simulations and a real data analysis that zero-filling exposure histories in DLMs can lead to biased estimates. This is because the practice induces a positive correlation between gestational age and exposures later in the exposure time series, which creates a positive confounding structure that was otherwise not present before. We also show that the bias is not confined to the zero-filled weeks, as the constraints put on the distributed lag function, typically through splines, can spread the bias into earlier weeks. This could explain why in many studies that zero-fill their exposure histories, the association appears protective (or trends in that direction) near the end of the lag–response [8–13].

In our simulations, we showed that conditioning on gestational age could potentially remove the zero-filled confounding. In our data application using Colorado birth data, adjusting for gestational age removed the implausibly strong association due to zero-filling, where all methods displayed similar lag–responses. But conditioning on gestational age also risks blocking the indirect effect through gestational age and inducing collider bias [15–17]. However, this did not appear to be a substantial concern in our data as the lag–responses were similar (for methods apart from zero-filling) when we did or did not adjust for gestational age, and when we restricted births to either 30 or 37 weeks. Furthermore, there are efficiency gains with gestational age adjustment, as it is a strong predictor of birth weight. Yet, gestational age adjustment may not always be the best option in other settings, where the choice to adjust or not should be informed by the several factors we discuss above including, but not limited to, potentially blocking an indirect effect (one should first check if exposure is related to gestational age); inducing collider-stratification bias if there are common causes of gestational age and birth weight (or one could use inverse probability weighting to circumvent conditioning on a collider [19]); removing confounding if there are common causes of exposure and gestational age; and increasing precision if the outcome is continuous (note that there are no gains for binary outcomes [20], such as low birth weight, etc.).

Bias due to zero-filling is a product of the investigator’s own making, and so we offer several alternative methods to handle exposure and gestational misalignment such as using their natural exposure, mean-filling, carry-forward, and truncating. These methods are expected to provide unbiased estimates, as they are not prone to the same type of bias inflicted by zero-filling since post-birth exposures are not dependent on gestational age. However, some of these methods may not be free of issues. For example, truncating the exposure window does not allow one to examine if late pregnancy exposures are associated with the outcome. In the Colorado birth data, the critical window appeared to be late in pregnancy, and so the other approaches (e.g. mean-filling, natural exposure) are more suited than truncation, especially since the presence of a late pregnancy exposures that are not adjusted for could cause bias in the estimated effect, which is likely why when truncating at 37 weeks, the end of the lag–response decreases more sharply than the other methods; and when truncating at 30 weeks, there appeared to be an early critical window (weeks 5–15).

In this paper, we only considered a linear DLM. For a distributed lag nonlinear model (DLNM), there is the additional concern of the basis expansion in the exposure direction (referred to as the “var” direction in the dlnm R package), where the choice of values used to fill the post-birth weeks can impact the basis expansion by affecting the placement of knots. If the values used to fill post-birth exposure weeks are within the observed range of the exposure data, they will only impact the internal knots. However, if the values fall outside the observed range—e.g. zero for PM_2.5_—it can also affect the boundary knots. These issues can be avoided by explicitly specifying the knot locations and by filling post-birth exposures with values within the observed range (e.g. carry-forward, mean-fill, natural through 42). Alternatively, truncating the exposure window at week 37 would also work in the absence of issues described above.

It is important to highlight that our simulations simplify the true causal structure—e.g. gestational age was simulated to be only a mediator but not a collider, and there were no competing biases (e.g. unmeasured confounding, selection bias, model misspecification). However, this was intentionally done to fully isolate the bias induced by zero-filling, though in practice we recognize that other biases could counteract or combine with the zero-filled confounding. In summary, the discussions here are meant to highlight the caveats of zero-filling and provide guidance on best practices for dealing with exposure and gestational misalignment in perinatal research.

## Ethics approval

This study was approved by the Colorado State University Institutional Review Board.

## Supplementary Material

dyag162_Supplementary_Data

## Data Availability

The code and exposure data are available without restriction at https://github.com/AnderWilson/ZeroFillDLM (DOI: 10.5281/zenodo.22033791).
