# Supplementary material for: Gestational misalignment with fixed exposure windows: the potential dangers of zero-filling in distributed lag models
Source: Int J Epidemiol. 2026 Sep 8;55(5):dyag162. doi: 10.1093/ije/dyag162 (PMC13553078; doi:10.1093/ije/dyag162)
Supplement: Supplementary file 1 [file dyag162_supplementary_data.pdf]

<u>Supplemental Materials</u>

Gestational misalignment with fixed exposure windows: the potential dangers of zero-filling in distributed lag models

Michael Leung, Andreas M Neophytou, Ander Wilson

Contents

Supplementary Sections

Supplementary Section 1	Description of Colorado birth registry data
Supplementary Section 2	Description of data generating scenarios for simulation

Supplemental Figures

Supplemental Figure S1	30-week threshold simulation results: time-varying association
Supplemental Figure S2	30-week threshold simulation results: time-varying coverage
Supplemental Figure S3	Colorado data analysis: time-varying association

Supplemental Tables

Supplemental Table S1	30-week threshold simulation results: bias, RMSE, coverage
Supplemental Table S2	Colorado data analysis: cumulative association

Supplementary Section 1. Colorado Birth Registry Data

The Colorado birth registry data includes all births reported in Colorado. We include only singleton live births with estimated dates of conceptions between January 2007 and December 2017, inclusive. We limited the analysis to the 13 Front Range counties and census tracts with elevation below 6000 feet. This area contains over 80 percent of the Colorado population and excludes areas with mountainous terrain for which exposure assessment is challenging. The final analytic sample size is 393,204. For the exposure data, we obtained $PM_{2.5}$ exposure estimates from the United States Environmental Protection Agency (EPA) community multi-scale air quality modelling system using down-scaled data[1], which were assigned to births by estimated date of conception and maternal residence at birth. For temperature data, we obtained the maximal daily temperature from the gridded surface meteorological dataset which contained the centroids of a 4-kilometer grid[2]. For both $PM_{2.5}$ and temperature, we calculated the weekly average levels for each census tract.

The figure below shows the distribution of estimated gestational age in the Colorado birth data. This distribution was used as a basis for the gestational ages in the simulation study.

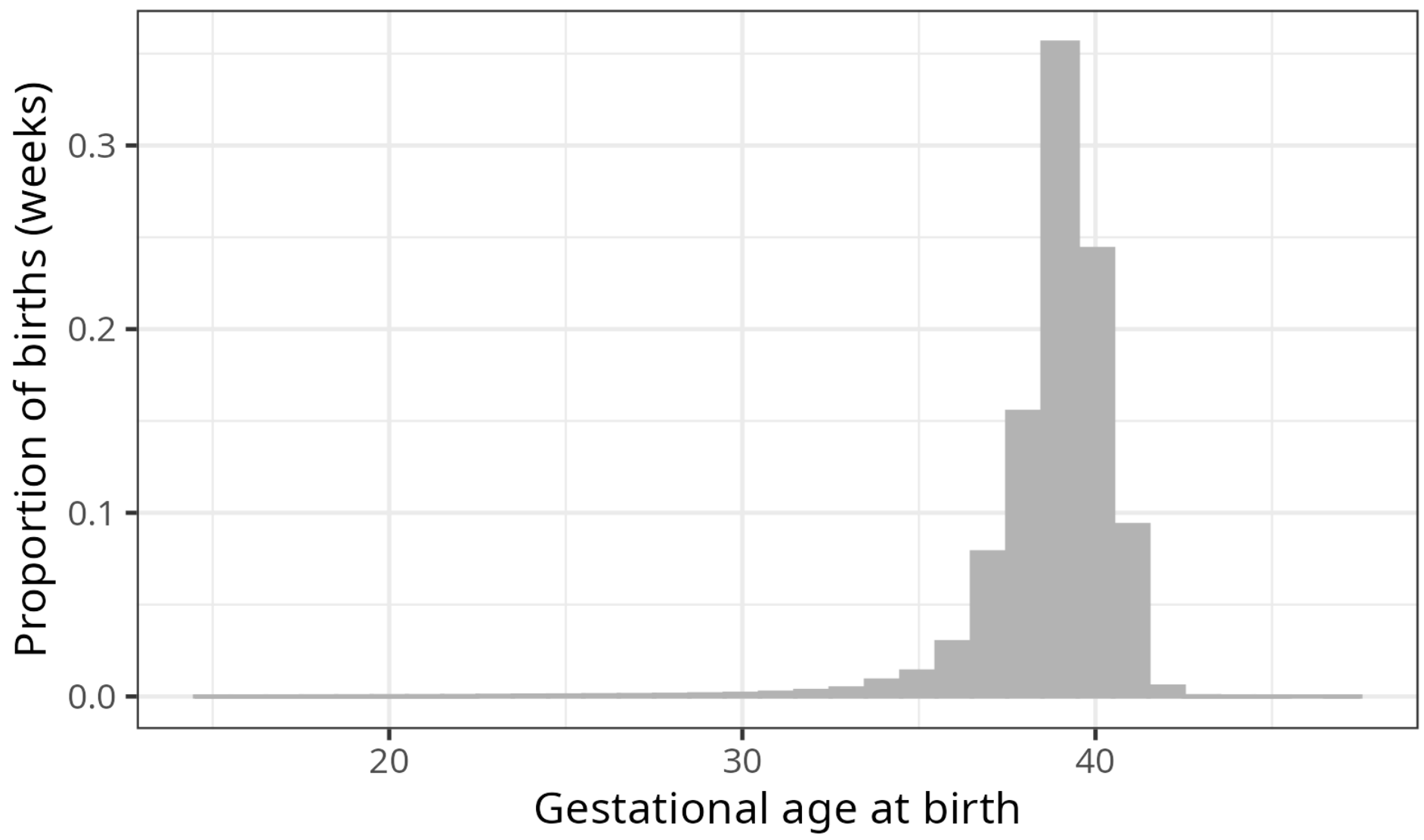

Information on several confounders for the association between air pollution and birth weight were also available including child sex, month of conception, year of conception, maternal age, income category, maternal education, indicator of prenatal care, maternal race, maternal Hispanic status, indicator of smoking, each trimester average temperature, county of residence, and elevation of residence.

Supplementary Section 2. Description of data generating scenarios for simulation

In scenario A, we randomly simulated gestational ages at birth following the empirical distribution in the Colorado birth cohort data from 37 to 42 weeks, so gestational age and birth weight do not share any common causes (unlike the DAGs in Figure 1). The negative effect of $PM_{2.5}$ was simulated to be between weeks 15 and 30, where the true data exposure-time-response function was $\theta_t = -1/[1 + \exp\{|22.5 - t| - 4.5\}]/8.96$ if t is between 15 and 30 and $\theta_t = 0$ otherwise. This gives a smooth, negative association peaking in weeks 22 to 23 with a cumulative effect of 1. We completed the design by adding random Gaussian noise with variance 25 and a linear effect of gestational age at birth with slope of 0.5 for every week greater than 37. In scenario A, we included no other covariate effects and no exposure effect following week 37 in the data generating mechanism so there can be no bias in the results due to those factors. Scenario B is the same as Scenario A except gestational age is simulated as a nonlinear function of average exposure between weeks 20 and 30 that results in a distribution similar to the empirical distribution of gestational age at birth in the Colorado birth data. As a result, gestational age is on the causal pathway between exposure and birth weight. Scenario C is the same as scenario A but has known confounders.

We include a linear effect of second trimester average temperature and a nonlinear seasonal trend using indicators of month of conception and year of conceptions. Note that this is slightly misspecified as the true effect is smooth and the month indicator is a step function approximation.

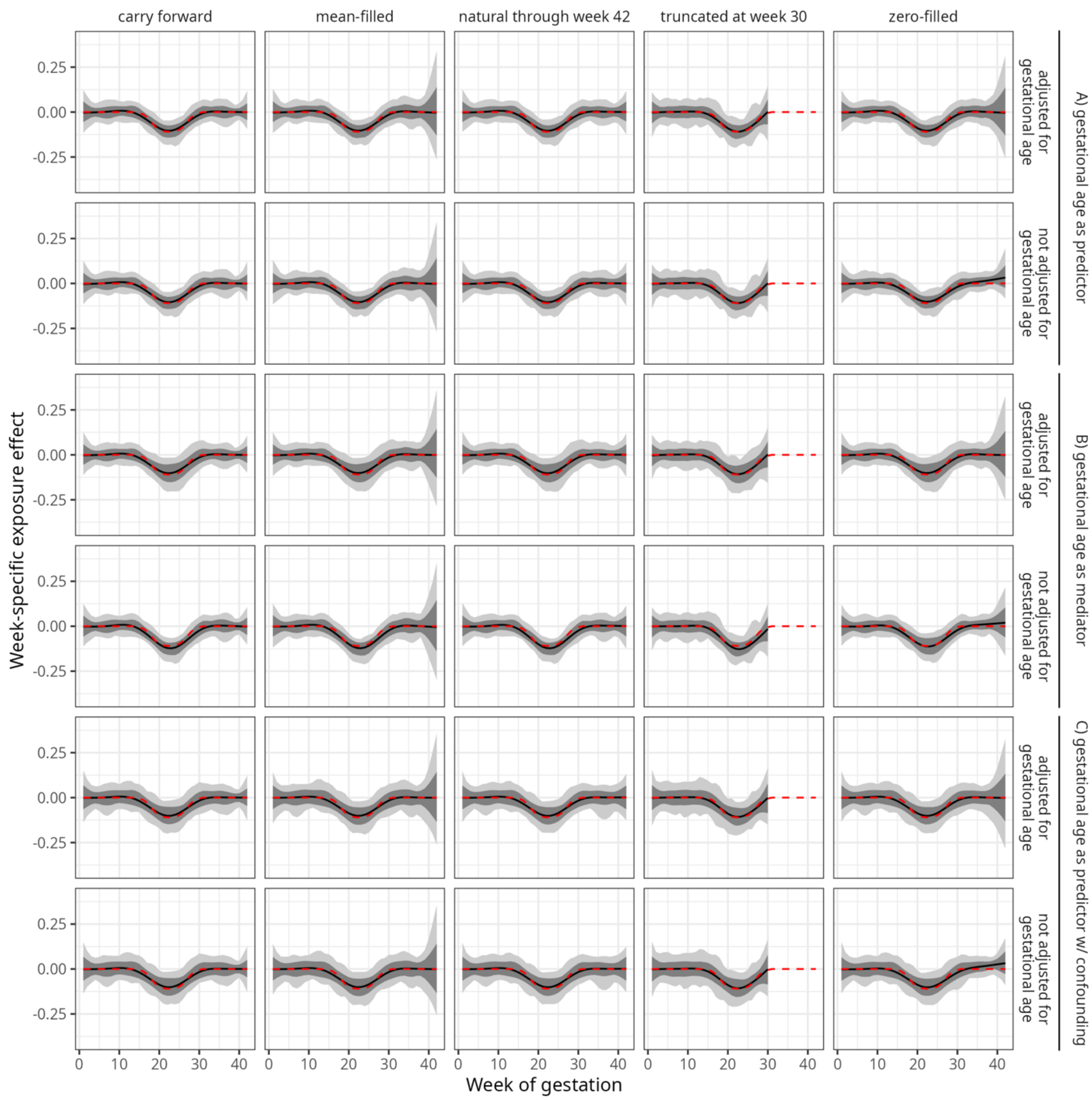

Supplemental Figure S1. Simulation results for with minimum gestational age set to 30 weeks. The figure shows the time-varying association between fine particulate matter ($PM_{2.5}$) and birth weight for five approaches to handling exposure and gestational misalignment (carry forward, mean-filled, natural through week 42, truncated at week 37 and zero-filled). Each column of panels contains results using one data method. The rows row of panels contains different scenarios and models adjusted for or not adjusted for gestational age at birth. The dashed red line indicates the true simulated lag-response relationship. The solid black line shows the mean of 2000 simulated data sets. The dark grey ribbon shows the 0.05-0.95 quantiles of the week - specific estimates and the light grey ribbon shows the minimum and maximum of the week-specific estimates. This is the same as Figure 2 of the main text except Figure 2 has a minimum gestational age of 37 weeks and this has a minimum gestational age of 30 weeks.

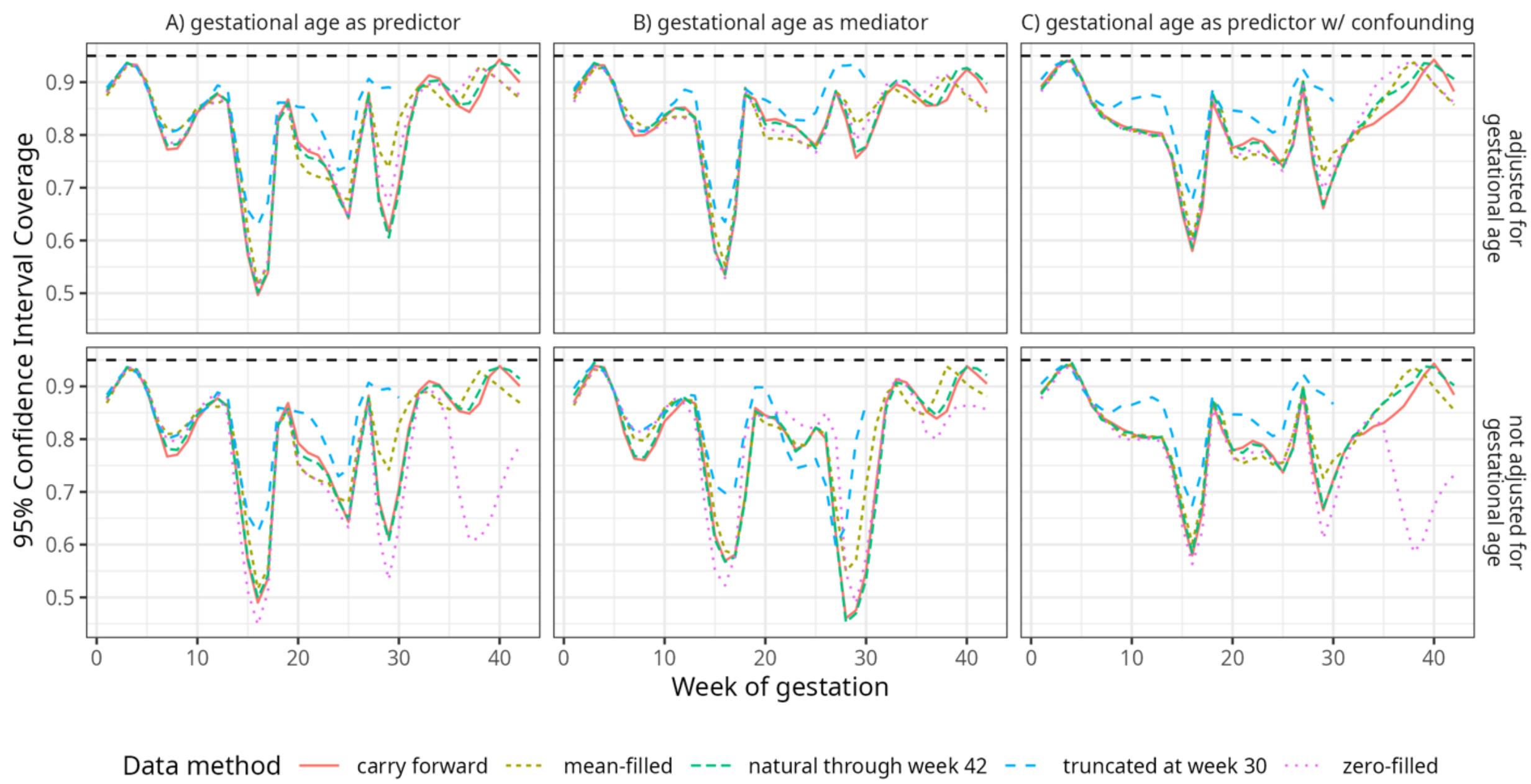

Supplemental Figure S2. Simulation results for with minimum gestational age set to 30 weeks. The figure shows the time-varying coverage between fine particulate matter ($PM_{2.5}$) and birth weight for five approaches to handling exposure and gestational misalignment (carry forward, mean-filled, natural through week 42, truncated at week 37 and zero-filled). The six panels represent three difference simulation scenarios and models adjusting for or not adjusting for gestational age at birth. Each line indicates the proportion of simulations in which the lag-specific 95% confidence intervals contained the true simulated effect for one of the data methods, and the black dashed line indicates the 95% nominal coverage. This is the same as Figure 3 of the main text except Figure 3 has a minimum gestational age of 37 weeks and this has a minimum gestational age of 30 weeks.

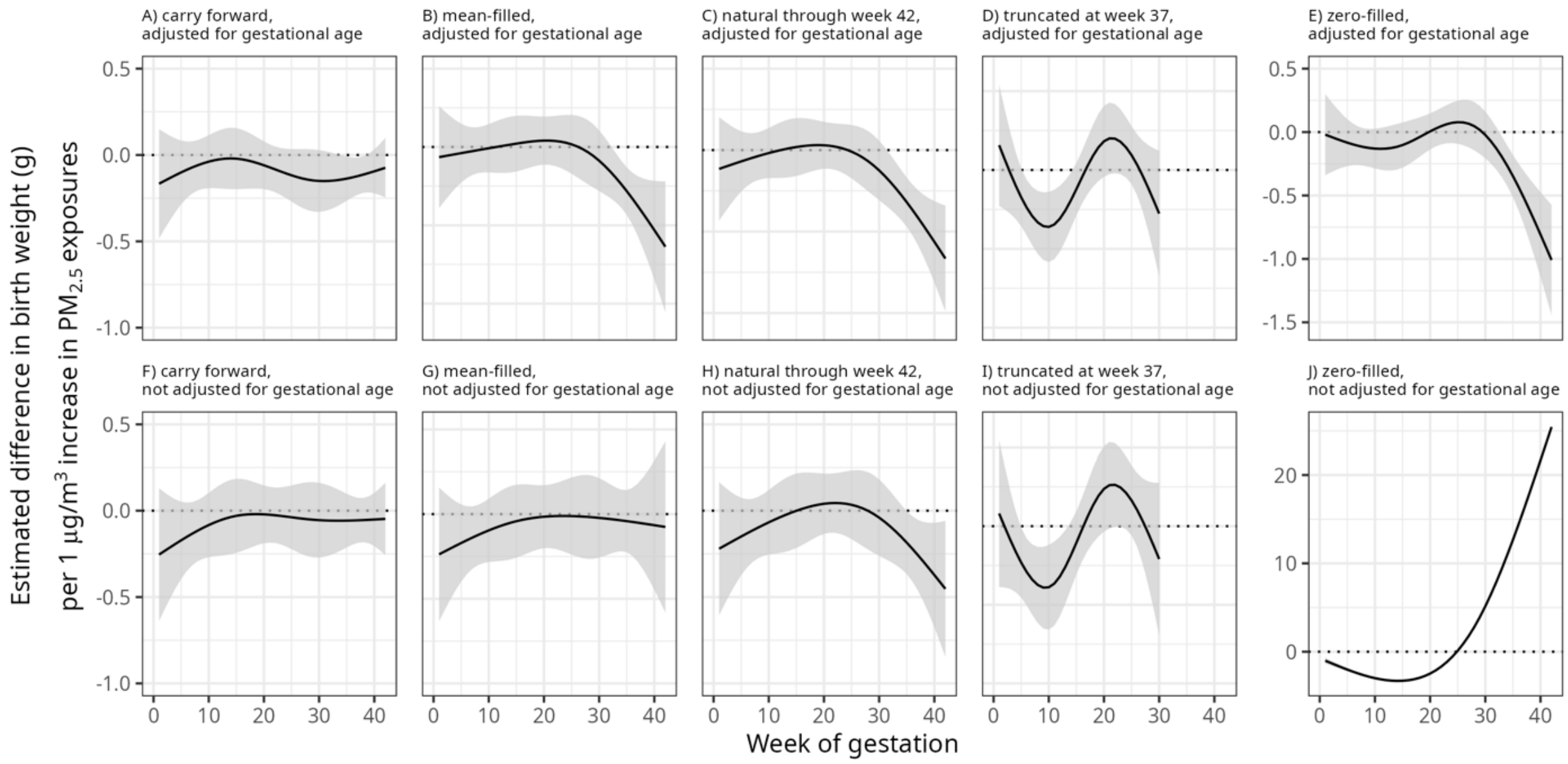

Supplemental Figure S3. Results from the analysis of birth weight in the Colorado birth registry data (*n*=393,204) with minimum gestational age set to 30 weeks. The figure shows the week-specific association between $PM_{2.5}$ exposure and birth weight using the five different data methods (columns) and adjusted for and not adjusted for gestational age (rows). The black solid line is the point estimate, and the grey ribbon is a pointwise 95% confidence interval. Panel J shows the estimates for the zero-filled data augmentation method without adjustment for gestational age and has a different y-axis scale than the other panels. This is the same as Figure 5 of the main text except Figure 5 has a minimum gestational age of 37 weeks and this has a minimum gestational age of 30 weeks and, therefore, a larger sample size.

Supplemental Table S1. Simulation results showing bias, root mean square error (RMSE), and percent coverage of the cumulative association between air pollution and birth weight for the different approaches to handling exposure and gestational misalignment. The true cumulative effect in the simulated data is 1. This is the same as Table 1 of the main text except Table 1 has a minimum gestational age of 37 weeks and this has a minimum gestational age of 30 weeks and, therefore, a larger sample size.

	Not adjusted for gestational age			Adjusted for gestational age		
	Bias	RMSE[a]	Cover.[b]	Bias	RMSE[a]	Cover.[b]
A) gestational age as predictor						
Carry forward	-0.003	0.029	95.1%	-0.003	0.029	95.2%
Mean-filled	-0.003	0.030	95.2%	-0.003	0.029	94.9%
Natural through week 42	0.000	0.031	94.8%	-0.001	0.031	94.9%
Truncated at week 37	-0.003	0.025	95.3%	-0.003	0.025	95.3%
Zero-filled	0.123	0.050	89.0%	-0.012	0.069	93.2%
B) gestational age as mediator						
Carry forward	-0.198	0.068	77.1%	0.015	0.069	93.3%
Mean-filled	-0.200	0.068	76.5%	0.019	0.070	92.9%
Natural through week 42	-0.191	0.067	79.6%	0.017	0.071	93.0%
Truncated at week 37	-0.199	0.065	75.0%	0.009	0.066	94.7%
Zero-filled	-0.030	0.094	91.6%	0.017	0.104	90.5%
C) gestational age as predictor w/ confounding						
Carry forward	-0.006	0.037	95.1%	-0.006	0.037	95.0%
Mean-filled	-0.005	0.037	95.2%	-0.006	0.037	95.4%
Natural through week 42	-0.003	0.038	95.5%	-0.004	0.038	95.4%
Truncated at week 37	-0.004	0.033	95.0%	-0.004	0.033	94.9%
Zero-filled	0.114	0.055	90.6%	-0.009	0.077	92.5%

[a] Root mean squared error
[b] 95% confidence interval coverage
[c] Performance metrics computed for the direct effect (i.e., not through gestational age)

Supplemental Table S2. Results from the analysis of birth weight in the Colorado birth registry data (*n*= 419,600) with minimum gestational age of 30 weeks. The table shows the estimated cumulative association (95% confidence intervals) for each data augmentation method and for adjusting for and not adjusting for gestational age at birth. The cumulative association is the expected difference in birth weight (g) per 1 $\mu g/m^3$ increase in $PM_{2.5}$ for all 42 weeks (or 37 weeks for the truncated model). This is the same as Table 2 of the main text except Table 2 has a minimum gestational age of 37 weeks and this has a minimum gestational age of 30 weeks and, therefore, a larger sample size.

Approaches	Difference in birth weight (95% confidence intervals), grams	
	Not adjusted for gestational age	Adjusted for gestational age
Carry forward	-3.04 (-6.45,0.37)	-3.92 (-6.73,-1.10)
Mean-filled	-2.81 (-6.24,0.62)	-4.53 (-7.35,-1.70)
Natural through week 42	-4.18 (-7.70,-0.66)	-5.56 (-8.46,-2.66)
Truncated at week 37	-1.92 (-5.15,1.30)	-2.25 (-4.91,0.41)
Zero-filled	143.75 (140.85,146.66)	-7.19 (-10.35,-4.03)

References

1. Berrocal VJ, Gelfand AE, Holland DM. A Spatio-Temporal Downscaler for Output From Numerical Models. *J Agric Biol Environ Stat*. 2010;15(2):176.

2. Abatzoglou JT. Development of gridded surface meteorological data for ecological applications and modelling. *International Journal of Climatology*. 2013;33(1):121-131.
